# Removal of Mesoblastic Tumor of the Neck

**Published:** 1896-02

**Authors:** S. H. Green

**Affiliations:** Camp Physician Chattahoochee Brick Company; Oak Dale, Ga.


### *CORRESPONDENCE.*

---

### REMOVAL OF MESOBLASTIC TUMOR OF THE NECK.

OAK DALE, GA., December 20, 1895.

*Atlanta Medical and Surgical Journal:*

On the 29th of October, 1895, I received into the hospital George Mason, yellow, sixty-seven years old, who had been suffering from a mesoblastic tumor for twenty years. On the 27th November, 1895, Dr. E. B. Bush, Principal Physician of the Penitentiary, and myself, operated on the patient and removed a tumor that weighed eight ounces. I dissected the tumor and found that it consisted of a dense mass of interlacing bundles of tissue, intermingled with a few yellow, elastic fibers of connective tissue capsules. The bundles in this tumor formed concentric circles around the blood vessels. But they presented no definite arrangements. I found a smooth, glistening, firm surface and a grayish white color. The blood supply was scant, the vessels being small and thin-walled.

This tumor was on the right side of the neck, involving the periosteum of the inferior maxillary bone. The muscles on the right side of the body were contracted so that the patient could not straighten up. For fourteen days before we operated he became paralyzed all over and could not move himself at all. But after we removed the tumor the patient was able to straighten up and sit erect in a chair, something he had not done in years. The wound healed up very readily under the antiseptic dressing of bichloride mercury, carbolic acid, and iodoform gauze with gum camphor and sweet oil. Patient improved nicely; appetite good, and was very fond of talking. I washed out his bowels every three days with warm water and castor oil, which moved them all right. Had to give him one-fourth grain of morphine and half glass of whisky every night to make him rest. He did not appear to be in very much .pain, said nothing hurt him, and he felt all right. I kept him on a good nutritious diet, and stimulated him with whisky and iron three or four times a day.

On the 4th day of December, 1895, the paralysis returned with greater force than ever. He lost his mind and became so violent that I had to keep two men by his bedside night and day. He continued to grow worse until December 7, 1895, when he died from heart-failure and paralysis. In my opinion, if we had been able to operate on him three months sooner, he would have recovered.

I have found from my experience that the sooner you operate the better results you will have. I have operated three times with similar cases and have had about the same results, owing to the fact that the patient waited too long before he would consent to an operation.

S. H. GREEN, M.D.,
Camp Physician Chattahoochee Brick Company.

N. B.: You must take into consideration the age of this patient and the length of time he had been afflicted with this tumor.

---

### LAPAROTOMY AND PENETRATING WOUNDS OF THE ABDOMEN.

Filippo (*Rid. Med.*, April 18th and 19th) reports a series of thirteen cases of abdominal injury with penetration in which laparotomy was performed. In six, in which the operation was merely exploratory, speedy cure resulted; in five, one or more wounds of the intestines or stomach were found, and of these one died, owing to the fact that one wound was overlooked at the time of operation. In two cases the left lobe of the liver was injured, and there was copious hemorrhage; of these, one is alive; the other died thirty-three days after operation from pneumonia. The author concludes that laparotomy, as a rule, gives good results in these cases, if done early. During the operation all the abdominal viscera should be most carefully examined, lest any small wound be passed by. Lavage of the abdominal cavity with some antiseptic useful. Subsequent ventral hernia not to be dreaded, whether incision is made *in situ* or in the linea alba. Suppuration of the abdominal walls is a not infrequent sequel after laparotomy for penetrating wounds.—*Brit. Med. Jour.*

